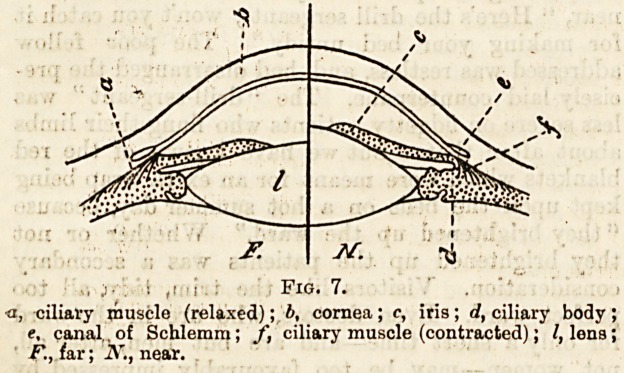# The Hospital. Nursing Section

**Published:** 1903-03-21

**Authors:** 


					tturotn* Section.
Contributions for this Section of " Thh Hospital" should be addressed to the Editob, "Ths Hobpiiai"
Nuksing Section, 28 & 29 8oathampton Street, Strand, London, W.O.
NO. 860.?VOL. XXXIII. 8ATURDA Y, MARCH 21, 1903.
motes on IRews from tbe mursina MorlO.
THE BEST SAVINGS BANK FOR NURSES.
The report of the proceedings at the annual
?meeting of the Royal National Pension Fund for
Nurses, which appears in our issue this week, will
he perused with extreme satisfaction by the numerous
?policy-holders among our readers. It will, we hope,
be carefully considered by those who are not policy-
'holders. There are many points of great practical
interest, but we especially direct attention to the
fact emphasised by the chairman, that the advan-
tages offered to policy-holders by the Fund are far
superior to those afforded by the Post Office Savings
Rank. In short, the Fund is itself the best savings
bank in the world for nurses, as the manager of
the Prudential Assurance Company, with his vast
experience, pointed out, while in the case of the
Post Office and other institutions the rates pub-
lished are for the actual amounts that will be
'Paid, no bonus being added, the figures given in
"the prospectus of the Royal National Pension
Fund are really the minimum sums that are
guaranteed, and in every instance there will be, as
?Mr. Dewey observed, an increased amount forth-
coming. This state of affairs is only rendered possible
by the voluntary services of the merchant princes
and the ablest financiers of London, who, because
they have the welfare of the nurses at heart, spare
no pains to obtain good investments for the Fund,
and whose labours of love find their reward in the
congratulations of the Queen and the knowledge
that they are crowned with splendid success.
MR. GRANT LAWSON, M.P, AND WORKHOUSE
NURSING.
If Mr. Grant Lawson, the Parliamentary Secretary
to the Local Government Board, has any voice in
determining the action of the Board with regard to
the report of the Departmental Committee on Work-
house Nursing, we are afraid that the prospect is
about as black as it could be. At the opening of
the new Workhouse Infirmary at Willesden, Mr.
Lawson having said that the report issued by his
committee had been criticised by people who had not
done them the honour of reading to the end of their
sentences, proceeded to make the astounding state-
ment that "in every particular " the Departmental
Committee "had tightened up the qualifications and
increased the status." Whatever may be the sins of
the critics of the report, Mr. Grant Lawson seems
hardly the proper person to criticise the critics, i If,
as it would appear, he thinks that the bestowal of the
title of " qualified nurse " on young women who have
had a year's training in a union workhouse infirmary,
is tightening up the qualifications, and increasing the
status, we can only deplore the fact that a man so
ignorant of the nursing question should have been
placed in a position of authority.
PROPOSED ? NATIONAL TRAINING SCHOOL FOR
MIDWIVES."
Lady George Hamilton and Miss Alice Gregory
have come forward with a proposal to build a
new general hospital with a maternity annexe in
one of the thickly populated outlying districts of
London, which they would have known as a national
training school for mid wives. We notice that accord-
ing to the scheme of these ladies, "educated women
would receive an 18 months' course of general; and
monthly nursing, prior to. a six months' course of
midwifery in hospital and district." But why 18
months 1 If educated women are to be trained in
general nursing, the period should be three years.
March 21, 1903. THE HOSPITAL. Nursing Section.. 337
We are in favour of the best possible training of mid-
wives, but we do not desire the creation of yet another
order of nurses who, while highly trained for mid-
wifery work, might, after their two years' course at
the proposed training school, take up other branches
for which they have not been adequately prepared.
THE MATRONSHIP OF WOODBRIDGE ISOLATION
HOSPITAL.
The new matron of the Woodbridge Isolation
Hospital must not be surprised if she finds that some
of the duties of her post are rather difficult to dis-
charge. We have before us a report of the pro-
ceedings at the meeting of the Guildford, Godalming,
and Woking Joint Isolation Board, at which Miss
E. A. Walker was appointed. There were five selected
?candidates, of whom two asked several very pertinent
?questions. For example, Miss Lanyon, who is at
present matron of the Lancaster Sanatorium, inquired
whether the matron had any control over the election-
-of the charge nurses. The clerk at once replied,
Oh no ; the election of the charge nurses has always
?been made by the Board, not by the matron ; in fact,
the whole of the staff is appointed by the Board."
Another candidate asked about the off duty time. A
member of the Board then proposed the election of
Miss Walker because, among other reasons, " she did
aiot ask what she could do and what she couldn't."
This argument appears to have been considered con-
clusive by the majority of his colleagues. Not the
least remarkable feature of the proceedings is that
immediately after the election of the new matron, the
Board occupied themselves for some time in altering
the rules under which she was appointed, the discus-
sion being marked at the close by personalties, in the
course of which it was asserted, without contradic-
tion, that the chairman and another guardian were
in the habit of taking tea with the nurses.
PLAYING AT NURSING IN PHILADELPHIA,.
It is stated in a New York paper that " a number
of wealthy young girls in Philadelphia have banded
themselves together to nurse the sick of the slums,"
that " they wear the ordinary uniform of the profes-
sional nurse, and are pledged to answer calls to
attend the sick at any hour of the day or night."
This seems at first sight a very nice arrangement on
the part of the wealthy young girls of Philadelphia ;
and it is interesting to hear that "over their spot-
less white uniforms they wear long coats," carrying
their caps in satchels " which also contain a number
of delicacies for the sick purchased by the willing
nurses." When, however, we come to the question
of the practical qualifications of these young ladies
for their self-imposed duties, we find that they are
merely taught " the art of nursing by competent
instructors, and that twice a week earnest young
students gather in the Witherspoon Building and
listen to lectures practically illustrated." This is
putting the cart before the horse. If there are
*l wealthy young girls" in Philadelphia who are
satisfied that their vocation in life is to nurse the
sick in the slums, their first duty is to undergo the
proper training for the work. After they have ob-
tained certificates of efficiency from a recognised
school! the spotless white uniform and satchel
containing delicacies will be unobjectionable, sup-
pdsing that the delicacies are given to patients with
medical authority;- - ::
THE "LADY DUDLEY" NURSING HOME IN;, ??
JOHANNESBURG.
Among the new institutions of Johannesburg the
"Lady Dudley" Nursing Home, so-called after its
patroness, is likely to be one of the most in request.!
It is situated in the most pleasant and open part of
Jeppe Street, and has accommodation for eight
patients?four single rooms and two with two beds
in each. The responsible heads of the home are
Miss E. Cockburn, of the Army Nursing Service
Reserve, who was trained at King's College Hospital,
and Miss G. A. Boyce, also of the Army Nursing
Service Reserve, who was trained at Guy's. Both
ladies served through the war, part of the time at.
Kroonstad, and the last eighteen months at No. 8;
General Hospital, Johannesburg, and at No. 13. The,
home is tastefully furnished, and the kitchen, which is,
outside, is presided over by, the lady housekeeper, -
who is a sister of one of the principals and does all
the cooking. She has been trained in cooking for
invalids. The electric light, mosquito curtains, and
an ice chest are among the comforts of the home.i
WARWICK GUARDIANS AND UNTRAINED NURSES.'
The Warwick Board of Guardians have happily
among them at least one lady who realises the
importance of employing fully-trained nurses in the
workhouse infirmary. Warwick has a new infirmary,
and it is proposed to have an adequate nursing staff.
But the ideas of the infirmary committee, whose
report on the question was considered at the usual
meeting of the board, show how difficult it is to get,
guardians generally to understand what an adequate
staff means. The committee recommended the;
appointment of a fully-qualified superintendent nurse
and of four assistant nurses, two with " at least six
months' training" and two who " should have had
experience in nursing." Mrs. Thursfield, however,
spoke out bravely against the employment of four
untrained nurses, which, she said, would be very
false economy, and strongly urged that " four nurses
with not less than three years' training should be
advertised for." While the Guardians were willing
to pay from ?24 to ?30 for untrained nurses, they
objected to the notion of paying ?26 and ;?30 with
?2 for uniform for trained nurses, and though the;
report was referred back to the committee, this step
was apparently taken because the board demur to
the appointment of a superintendent nurse.
SAD DEATH OF A DISTRICT NURSE.
The death of Miss Kate Catchpole, who threw her*-
self from Waterloo Bridge recently, was attributed
by the jury to suicide while temporarily insane. The
unhappy young woman, who Was thirty years of age,
came up to London, after nursing on her own account
a private case at Gorleston, on March 9thj and ended
her life about midnight on the same date. It was
stated at the inquest that she had suffered a good
deal in her head, but no light was thrown upbn her
motive. Miss Edith Watson, Lady Supdrintehdent
of the Norfolk and Norwich Staff of Hospital Trained
Nurses, with which Miss Catchpole was about to
becbme associated, says that" the course she took is in-
explicable to us all." Miss Watson thinks that her
strength had been overtaxed when sh^ was & district
nurse at Greenwich, and* thatshe :had not: fully
recoveredj although the brading air b? the Eastern
Counties, whichf she .had; been a^vdsedj to jtryf had
338 Nursing Section. THE HOSPITAL. March 21, 1903.
seemed to benefit her. Two light cases were given
Miss Catchpole to test her fitness for acceptance on
the Norfolk and Norwich staff, and both the lady
superintendent and the patients were pleased with'
her. She was trained for district nursing at St.
Mary's Hospital, Paddington, and in July and
August 1899 at the British Lying-in Hospital,
Endell Street, and had been a nurse for some years.
The sad occurrence has been a great shock and
trouble to all the staff at the Nurses' Home in
Norwich.
AN EXPERIMENT IN CANADA.
i Under the sanction of the Canadian Government,
a British settlement is about to be founded in
Sasketchewan, North-Western Canada, and 2,000
persons have booked their passages in the steamship
Lake Manitoba for Wednesday next. It is expected
that some 20,000 will settle there within ? the next ?
twelve months, and townships will be founded on
virgin soil. The Rev. I. M. Barr, the originator of
the enterprise, has decided that the interests of the
colony require a hospital with a trained nurse at its
head, and he has accordingly appointed Miss James,
charge nurse at the Gordon Hospital, Yauxhall
Bridge Road, to go out as matron. Miss James
has had about nine years' hospital experience;
she was trained at the Western Infirmary, Glasgow,
and was subsequently charge nurse at the Fountain
Fever Hospital, Tooting, and at the City Hospital,
Coventry. She has been two and a half years in her
present position. The task of buying the entire
hospital outfit in London has been entrusted to her,
as will be the gathering around her in Canada of
suitable helpers for the hospital. Immediately on
arrival at Saskatoon, where the railway is exchanged
for a " trek " by waggons, a halt will be made, and
the hospital will be set up. This will consist of
12 beds under canvas, which will be ready for use
day by day on the way. At the journey's end a
permanent hospital will be erected, with a separate
building for contagious diseases. "The hospital,"
says Mr. Barr, " will have an open door, where the
best of nursing and medical attendance may be had,
under the best sanitary conditions, and amid com-
fortable surroundings."
thirty hours on duty.
( . - . , ,
An irresistible case for an addition to the nursing
staff at Wirral Union Infirmary was made out by
Mrs. Hodgson at the last meeting of the Wirral
Board of Guardians. Mrs. Hodgson stated that
there were 47 cases in the infirmary, of which 18
were bedridden, eight suffering from senile decay and
requiring attention night and day. For eight wards,
she continued, there were two nurses and two pro-
bationers, whose hours of work were from seven in
the morning to ten in the evening. In these circum-
stances we are not surprised to learn that by ten
o'clock the hands and feet of the nurses are weary,
and Mrs. Hodgson affirms that " under the present
arrangements work begins afresh for the nurse on
night duty." She gave an instance of a nurse who
had been on duty altogether for 30 hours, and added
that " this happened frequently." Mrs. Hodgson
was followed by Mrs. Dalglish, who submitted a
statement from one of the nurses showing that, " for
?nearly a fortnight the two nurses had, in addition to
;th6ir work during the day, stayed on at night alter-
nately, the probationer being too inexperienced to
perform the work." Action was deferred by the
Guardians until the doctor's report had been
obtained ; but, assuming the accuracy of the allega-
tions of the lady members, the appointment ofi
another nurse for night duty is an urgent necessity.
COLONIAL TRAINING FOR NURSES.
It will interest nurses who think of going out to
South Africa to learn that there has been started at
Swanley Horticultural College in Kent a Colonial'
branch, intended to afford a short course of training
calculated to fit intending women workers for colonial
life. Instruction is given in simple cooking and
housekeeping, laundry work and dressmaking, the
care of poultry, etc. Many nurses who have been in
South Africa can testify to the difficulties which they
encountered on arrival. Professional capacity is
highly valued, but nurses must be able to turn their
hands and minds to other things than nursing onlyr
and if women go out there unprepared to do this
they will have a hard time before them. The charges
at the branch, which is known as West Bank, are
moderate, and in addition to the training given,,
pupils can, for small extra fees, take short courses of
instruction from the college lecturers. At the end of
a course of one term, a recommendation, according
to proficiency and conduct, will be given by the
College, and the pupil put into communication with'
colonial openings. After a year's course an examina-
tion will be held, and a diploma awarded.
NURSES IN AMERICAN SCHOOLS.
The Health Department of New York has ap?
pointed nurses to 39 public schools in Manhattan.
They are required to examine the children as to
cleanliness and to instruct the mothers at their
homes how to treat all cases of sickness. Principals
are asked to render the nurses as much assistance as
possible; and the innovation has been generally
welcomed by the school authorities.
A SUCCESSFUL LANCASHIRE ASSOCIATION.
The twenty-fourth annual report of the Wootton
District Nursing Society is very satisfactory, both as
to the amount of work done, and also as to the finan-
cial position. In 1902 the nurses attended 282
patients and paid 7,166 visits. The income for the
year was ?220 8s. 6d., and the expenditure
?200 0s. 3d. An interesting feature of the report is
the statement that Nurse Key completes this year
the tenth of her connection with the society, during
which period the report says she has endeared her-
self to her patients and gained the esteem of the ?
committee and of those associated in the work.
SHORT ITEMS.
The King has bestowed the decoration of the
Royal Red Cross upon Miss M. Nicholson, Miss
C. E. Thompson, and Miss S. M. G. Otto in recogni-
tion of the services rendered by them in tending the
sick - and wounded at the Volunteer Hospital afe
Intombi during the late war in South Africa.?At
the West Ham and East London Hospital, last week,
an examination was held for senior probationers on
the lectures delivered by the matron and house
surgeon. The examiners, the honorary medical
staff, passed all the eight probationers, awarding the
first prize, given by the General Committee, to Nurse
Waller, and the second to Nurse Morten.
March 21, 1903. THE HOSPITAL. Nursing Section. 339-
Zhe nursing .?utlooft.:
" Prom magnanimity, all fear above;
From nobler recompense, above applause,
Which owes to man's short outlook all its charm."
"BRUTAL" NURSES.
Colonel J. Elliot, of Toronto, complains indig-
nantly of what he calls the " brutality of many trained
nurses." He says of these :?" First, they get trained
into them a certain amount of medical skill, shaking
up pillows, taking temperature, pulse-feeling, and
keeping records of the latter; secondly, they get
trained out of them all the attributes which God has
placed in the soul, heart, and mind of man and woman
(especially in the latter), such as love, tenderness,
gentleness, and such like ; thirdly, in the place of
these Godlike attributes, they have trained into them
callousness, coldness, indifference to the sufferings
of others, independence, rudeness, impatience,
self-opinion, will power in a wrong direction, and
every other such lit e opposite to the better part
of a human being." This conviction as to the charac-
teristics of trained nurses comes, the Colonel says,
from personal experience, and he includes both
English and Canadian nurses in his condemnation.
This is a very serious indictment, and, for our own
part, we agree with the editor of Public Opinion
"who, in publishing the letter, adds that the
gallant colonel " overstates a case that, in the
opinion of a good many people, is sufficiently serious
if put with all possible reserve." That in the opinion
of a good many people there should be any appear-
ance of foundation for such accusations being brought
against trained nurses is quite bad enough. We
believe that there is some reason for the complaint,
and yet that the nurses are not seriously to blame.
The complaints against nurses come as a rule from
private patients, and private nursiDg is so different?
not in essentials but in little marks of consideration?
from hospital nursing, that the one is by no means
a perfect preparation for the other. For this reason,
the principals of many nursing homes prefer to train
their own nurses. Hospital training does not inevit-
ably teach that endurance of the patient's whims
and caprices, that willingness to make trivial con-
versation or read a book in which one is not inter-
ested, or in many ways show that self-effacement in
trifles which is demanded of the private nurse.
Many nurses grow impatient and forget that
whereas in the case of many hospital patients,
the conditions of life in hospital are eo much
better than those of their homes, that in themselves
?hey have a curative effect, and they require no special
attention or petting from tlie nurse?which indeed in
her laborious day she has little time to give, a private
patient finds illness, with its accompanying weakness
and pain, a bitter interruption to ^ life which is com-
ortable and sometimes luxurious, &nd requires to be
amused and comforted almost like a\child. The nurse
resh from hospital does not see the i)eed for giving
and is often looked upon as unsympathetic and
n eeling, when at the worst she is\ simply dull.
eople have not yet got over?and it t^iil be a bad
day for the nursing profession if they ever do get
over?the notion that nursing is a work which is
taken up from a higher feeling than the mere need
of making a living. They expect more of personal
service and tenderness from a nurse than they do
even from their doctor. Yet it is easier for the
doctor to listen with the appearance of attention for
a few minutes than for the nurse to sympathise
every hour of the whole long day. Let us fully
admit that patients are often unreasonable. That
does not affect the fact that no woman should
take up the work of nursing who is not prepared to
put into it a great deal of personal devotion, patience,
and self-sacrifice. Nursing, taken up merely as a
trade, loses one-half of its good. That some nurses
come out at the completion of their training with &
perfect knowledge of the technique of their work,
but lacking any comprehension of its spirit is unfor-
tunately true. Even in hospital, consideration for
the individual patient might sometimes, with ad-
vantage, takethe place of consideration for the appear-
ance of the ward. A nurse once told the present-
writer that she received a lesson she never forgot by
overhearing one patient say to another as she came
near, " Here's the drill-sergeant; won't you catch it
for making your bed untidy." The poor fellow
addressed was restless, and had disarranged the pre-
cisely-laid counterpane. The "drill-sergeant" was
less severe on fidgetty patients who flung their limbs
about after that. But we have known of the red
blankets which were meant for an extra wrap being
kept upon the beds on a hot summer day, because
" they brightened up the ward." Whether or not
they brightened up the patients was a secondary
consideration. Visitors like the trim, tidy, all too
perfect ward. Even doctors, who are in the ward'
for only a short time?and are but men, after all,
not women?may be too favourably impressed by
this exquisite neatness, and unless the matron be a-
truly wise and sympathetic woman, who can put
herself in the place of the patients, Nurse So-and-so
may be marked for promotion because her ward is
always in such order, and her patients as afraid to
move as a regiment on parade. In which case one
may be sure that Nurse Somebody-else will try to
win promotion in the same way. So grows up in the
whole staff the notion of patients as beings who are
to be not merely controlled and disciplined, which is
often necessary, but domineered over. And this?
to put the matter on no higher grounds?is very bad
training for private nursing, where the patient is not
only sure to resent such treatment, but has the
power to make his resentment felt. This is at least
one reason why some people declare their preference
for the old-fashioned, untrained, and perhaps un-
skilful nurse, who is yet a kindly woman, and their
dislike of the trained product of the modern hospital.
We are far from saying that mere good nature takes
the place of scientific training. But, on the other
hand, scientific training should not be allowed to
take the place of kindliness. That many nurses have
all their womanly tenderness " trained out of them
we do not believe, but some have it so much overlaid
by the habit of ruling as to give some ground for
Colonel Elliot's protest, even though it is true that,
as the editor of the journal which prints his com-
plaint declares,. and as we are sure, he grievously
overstates his case.
340 Nursing Section. THE HOSPITAL. March 21, 1903.
lectures on ?pfttbalmic IRursfng.
By A. S. CobblediCk, M.D., B.S.Lond., Senior Clinical Assistant Royal Eye Hospital, late House-Surgeon and
j ' Registrar, Royal Eye Hospital.
LECTURE VI.?ACCOMMODATION?FUNCTIONS OF
THE IRIS AND RETINA.
The most important of the three changes which occur
in the eye during accommodation for near objects is the in-t
crease in the curvature of the anterior surface of the lens.
When the eye is in its natural condition of rest there is an
agency at work which keeps the anterior surface of the lens
flattened: this agency is the suspensory ligament of the
lens, which has been previously described. When accom-
modation for near objects takes place, the suspensory liga-
ment slackens and the inherent elasticity of the lens asserts
itself, with the result that the antero-posterior diameter of
the lens is increased. The question is, What produces the
slackening of the suspensory ligament ? Experiment has
shown that the ligament is slackened by the action of the
ciliary muscle, which, when it contracts, draws the choroid
coat forwards, renders the ciliary body more loose, and there-
fore gives the ligament more play.
The following diagram (after Helmholtz) shows one-half of
the eye accommodated for near and the other half accom?
modated for far objects.
The contraction of all the muscles concerned in the act of
accommodation, viz, the ciliary muscle, the internal recti,
and the sphincter iridis, are under the control of the third
cranial nerve.
Movements of the Iris.
The iris contracts, i.e., the pupil becomes small?1. On
exposure to light; 2. On accommodation for near objects;
During sleep; 4. Under the action of certain drugs, e.g.
eserine or opium; 5. When, from any cause, the third cranial
nerve is irritated; 6. Paralysis of the cervical sympathetic.
The iris dilates, i.e., becomes larger?1. In the dark;
2. When the accommodation is relaxed; 3. Under the in-
fluence of certain drugs, e.g., atropine and chloroform;
4. Under the emotion of fear and during severe pain ;
5. When the third cranial nerve is paralysed; G. When the
cervical sympathetic is stimulated.
The most frequent and common cause of movement of the
iris is exposure to light.
This is another instance of a reflex action; the light rays
impinging on the retina stimulate the optic nerve fibres, which
form the afferent or sensory nerve; the centre is in the region
of the corpora quadrigemina, which in turn is connected by
fibres (Meynert's fibres) with the third cranial nerve nucleus'.
The third cranial nerve whiclj supplies the iris is the efferent
nerve. ,
With regard to drugs which act on the iris, it may be said
that those which dilate the pti^ll are bailed mydriatics, and
those; such as physostymine, of its active principle eserine,
which contract the pupil are termed myotics.'
Drugs may produce alteration in the size of the pupil if
taken internally or if applied locally. Most of those used in
ophthalmic practice act locally, e.g., atropine paralyses the
sphincter iridis and ciliary muscle when all the nerves to the
eye have been divided.
The dilatation produced by chloroform and the action of
intense emotion is through their action on the centre in the "
brain.
The iris by these acts of contraction and relaxation serves
some important purposes:?
1. The contraction on exposure to light regulates the
amount which enters the eye.
2. It acts as a diaphragm so as to lessen spherical aberra-
tion, i.e., it does not allow rays of light to pass through the
lens near its periphery. Generally speaking, an object is
most clear and distinct when the pupil is contracted, and
becomes blurred and indistinct as the pupil dilates, e.g., after
the action of such a drug as atropine.
This action is also aided by the dense pigment on the
posterior surface of the iris on the ciliary processes and the
back of the retina, which absorbs the greater part of the
light that is reflected on to the eye.
The movements of the iris are governed by the third
cranial nerve which supplies the sphincter iridis; the
cervical sympathetic, which supplies the radiating fibres;
and fibres of the fifth cranial nerve, which are sensory.
The two centres in the brain that govern the movements
of the two irides are closely connected, e.g., if one eye is
shaded with the hand the pupil of that eye dilates, but the
pupil of the other eye also does the same. The same remarks
refer to contraction.
Functions of the Retina.
The retina is a thin membrane separated from the vitreous
by the hyaloid membrane, and firmly attached on its outside
to the choroid ccat. It is a very important structure, as it
is almost exclusively composed of the fine termination of
the optic nerve; that is to say, it is a layer of specially modi-
fied nerve fibres and endings.
If a section of the retina is examined under the micro-
scope, it is found to consist of as many as 10 distinct layers,
viz.:?1. An internal limitiog membrane; 2. A layer of optic
nerve fibres. This varies in thickness, becoming thiner?as
one might expect?towards the anterior portion of the retina;
8. A layer of ganglion cells; 4. Two molecular and two
nuclear layers; 5. An external limiting membrane ; G. A
layer of rods and cones; 7. Layer of pigment cells.
The layer of rods and cones is a very important one, as
each rod and cone is a termination of an optic nerve fila-
ment. The rods are more numerous than the cones, except-
ing at the fovea centralis, where cones only are present.
Since the fovea is the point of most acute vision and also
the most sensitive part of the retina to light, it is highly
probable that the cones take a more important part in vision
than do the rods.
Notice that when li^ht impinges on the retina it has to
pass through all the layers before it reaches the rods and
cones. I
At the point of entrance of the . optic nerve, i.e., at the
optic disc, there are no rods aiid cones, and hence it is quite
insensitive to light: it is called the blind xpot.
The presence of the blind spot may be easily made
manifest by the following experiment:?
^ +
Close the left eye and look steadily at the black spot at ti
distance of about 6 inches ;? then gradually increase th'o
distance between the eye and | the spot by removing the
farther from .the face ; a point will be Reached where
the cross disappears from view, but.it soon cpmes, into view'
again on still further increa^ihg the distance. i. "
J ?
,i ' i . : Fig. 7.
a, ciliary muscle (relaxed); If, cornea ; c, iris; d, ciliary body ;
e, canal,, of Schlemra; f, ciliary muscle (contracted); I, lena;
F., far; N., near.
March 21,' 1903. THE HOSPITAL. Nursing Section. 341
IRopal national pension tfunb for nurses.
The sixteenth annual general meeting of the members of
the Koyal National Pension Fund for Nurses was held at
River Plate House, Finsbury Circus, London, on Thursday,
March 12th, 1903, under the presidency of Sir Henry
Burdett, K.C.B. >? >
Supporting the chairman were the following members of
council:?Mr. Edward Eawlings, Mr. R. Ernest Alexander,
Mr. Thomas Charles Dewey, F.I.A., Mr. Charles W. Trotter,
and Mr. Falconer L. Wallace. Amongst those present were
the Hon. Herbert C. Gibbs, Sir Cuthbert Quilter, Bart., M.P._,
Mr. F. D. Mocatta, Mr. Perceval A. Nairne (Chairman Sea-
Man's Hospital, Greenwich), Dr. George W. Potter, Mr. A. G.
Waley, the Sister Superior (Nursing Sisters of St. John the
Divine), Mrs. Lewis Ogilvy (East London Nursing Society),
Mrs. Pritchard Binnie (Hon. Secretary of the Junius S.
Morgan Benevolent Fund), Colonel George Stockley (Kent
Nursing Institution), Mr. Conrad W. Tbies (Secretary Royal
Free Hospital), Miss Mabel. Cave; (Matron Westminster
Hospital), Miss P. Peter (General Superintendent Q V.J.I.),
Miss S. A Swift (Matron Guy's Hospital), Miss E. Vincent
(late Matron of St.' Marylebone> Infirmary), Miss Oxford
(Lady Superintendent Guy's Trained Nurses' Institution),
Miss M. Emerson (the London Hospital), and a large number
?f policy-holders.
The Secretary (Mr. Louis H. M. Dick) having read the
notice, i t v
The Chairman said: Ladies and gentlemen, letters of
regret at their inability to be present have been received
from Mr. Thomas Bryant, F.R.C.S., the Hon. Egremont J,
Mills, Mr. Eric Hambro, M.P., and a great many others. I
have now to move the adoption of the report, which has been
printed and circulated, and a copy of which is in your hands.
You will have observed that the growth of the Fund has been
very satisfactory. The pension policies have increased from
842 to 869, and the total policies from 8,942 to 9,811. The
sick pay policies have increased from 2,476 to 2,585.. The
actual funds have increased from ?648,700 to ?734,000, and
those of the i Junius S. Morgan Benevolent Fund from
^18,396 to ?20,285. I think it may be interesting to compare
the growth of the Fund during the last 10 years. In 1892
the pension policies issued were 486, and in 1902 the busi-
ness had so increased that they had risen to 869 for that
year. The nurses entitled to sick pay in 1892 were 392, and
this year they have increased to 1,568. The premium income
|Qirl892 was ?2(5,580, and this year it was ?94,648. The
income from investments in 1892 was ?4,496, and to-day it
is ?27,000. The total income, which in 1892?10 years ago
?was ?31,000, is to-day ?121,568 per annum.
A Savings Bank. ? ;
In connection with this Fund we have a savings bank
branch, which is available for nurses under the terms of
their policies. They are enabled to save their money by
taking out a policy in the Pension Fund, and they have the
^Sht if they wish to do so, to surrender those policies
and take out the premiums which they have paid in,
with compound interest at the rate of 2^ per cent, per
annum, or to withdraw a portion of their money only.
As showing the immense value to nurses of this feature
the Fund I " may mention that during the year
^902 they took out of the Fund, as accumulated savings,
^-5,000; and those who know most about nurses and you
nurses who are present will, I am sure, agree with me?
regttrd it as a fact that had it not been for the Pension Fund
^tfy little, if any, of that ?25,000 would in all probability
have been eaved, nor would it have been available for the'
nurses for their own' purpose? when they decided to withi.?
draw it from:the Pension Fund. As to.the actual work
done for nurses in the fulfilment of the objects of the If unci
it is satisfactory to report that whereas 10 years ago the
actual money paid in pensions to nurses was only ?235, this
year it has increased to ?8,000 per annum. That is to say
that the nurses who have finished their work and retired
are at present receiving ?8,000 a year in pensions from this
Fund. Turning next to the working expenses and calcu-
lating them on a percentage to premium income, we find
that whereas at the end of . the first quinquennium, in
1892, the expenses were 6|- per cent., at the end of the
second quinquennium, in 1897, they had fallen to a little
under 4 per cent., while at the present time, the end of the
third quinquennium, they have been reduced to a little over
3? per cent. The funds at the present time exceed ?750,000.
The Invested Funds and the City.
With reference to the investments, I am sure it will be satis-
factory for you to hear, although it,will not surprise you when
you know it, that through the voluntary services of the merchant
prinees of London and of the chief financiers of the City?
for there is no one in the City of London who will not give
his services and take trouble to secure a good investment for
this Fund?the appreciation on the whole of our securities,
despite the somewhat hard, times which investments have
had to go through in recent years, amounted to 2 per cent,
on their capital value at December 31st last. I consider
that no words of mine and no testimony could be more
eloquent to the valuable services rendered to nurses by tbe
City of London in connection with our affairs than that fact;
and if we turn to the Benevolent Fund, which is an added
interest to the Society, and especially an interest to the house
of Morgan?for it was founded to commemorate that great
and good man,, without whom, probably, we should npver
have had this Fund, the late Mr. Junius S. Morgan-r?it is a
special pleasure to me to be able to state that the invest-
ments of the Benevolent Fund have appreciated 7 per cent.
The average rate of interest which we are able to earn
through the co-operation of the gentlemen to whom I have
referred, amounted this year to ?4 2s. 9d. per cent.; and I
see that Sir Cuthbert Quitter is pleased at that statement.
He knows whas that represents, for it is not an easy thing
nowadays on approved securities to earn over 4 per cent,
or even 4 per cent, at all; and yet you must know that an
average rate of over 4 per cent, means a larger amount for
profit interest, a considerable addition to your bonus fund.
Nurses and Sick Pay.
Turning next to the sick pay, last year 15 nurses re-
ceived sick pay during every week of the year, and they
had collectively divided among them ?523 out of the
total amount distributed of ?1,568. Of the 254 policy-
holders who received sick pay, some came on the Fund more
than once during the year, and the whole number o!f separate
illnesses amounted to 325. I should like to say with respect;'
to the sick pay, that I do not think that nurses nowadays
appreciate how important it is to them when they join the
Pension Fund not only to enter for a pension, but also for
sick pay. The premium upon the sick-pay policy is a rela-
tively small matter in view of the whole premium for pension-
and sick pay; and there is no doubt, having regaird to the
risks to health which are daily and hourly incurred neces-
sarily by nurses in the pursuit of their avocation, that those
who are wise and prudent, and those who have; the best/
heads for business among the nurses, will take very great
care that they do nob allow the safeguard and security of the
sick-pay fand to pass them, and that when considering what*
form of policy they will take out they will turn to those,
pages in our tables wliioh provide that under a composite!
342 Nursing Section. THE HOSPITAL. March 21, 1903.
ROYAL NATIONAL PENSION FUND FOR NURSES ?Continued.
policy they axe entitled to receive not only a pension at a
given age, but sick pay also whenever they need it, and sick
pay continually from the time they need it until they are
able to resume their duties. That is a most important
?feature of this Fund. It is one to which I personally
attach the utmost value, for this reason, that if you look
at the occupation of a nurse and realise all the risks which
occur to her especially?which do not apply in regard
to the ordinary population?I am quite confident that no
fund aiming at pensions for nurses would be adequate to
fulfil all that was required of it unless it could say to every
member, as this Fund can say, " If you join this Fund and
join it for sick pay also, you may rest assured that you
can devote all your energies to your work without any
anxiety as to your future, because you know perfectly well
that providing your life is A1 in the insurance sense, what-
ever may befall you, under a composite policy for pension
and sick pay you will be secure, with the assistance of this
Fund, for all your life."
Nubses Help Yourselves and the Fund.
I should like next to deal with this Fund as a mutual
fund. I do not think it is sufficiently understood among
the nurses that those who join the Fund can render an im-
mense service to each other by doing their utmost to uphold
its credit, to answer objectors, and at all times to make it
known among their fellow-workers, so that we may increase
as far as possible the usefulness of this Fund by adding to
the number of policy-holders. Many of you know that
there are all kinds of objections urged why a nurse
should not join this Fund. One very favourite objection is
that the rates are too high. That was an objection which
was raised against the Fund when it was started, and some
of the most eminent authorities in the hospital world
told me?and I have their letters now?that it was quite
impossible to build up a Pension Fund for Nurses?that
they could not afford to save, that they had not the
means to provide for themselves, that the claims upon
them were too numerous, and that it was hopeless to ex-
pect that the Fund could succeed. That was what was
said when we started it. Well. I think the best answer
to all that is that at the present time we have some
thousands of nurses who are members of this Fund, and
that they are paying in out of their earnings nearly ?100,000
per annum. It is therefore quite clear that everybody who
wishes to join the Fund, who wishes to be provident, who
wishes to take the safeguards which this Fund offers, can,
being a nurse, do so perfectly readily, and that the scope of
<the Fund is well within their possibilities. Another very
?favourite objection is that when the nurse gets qualified
?and receives her certificate she is too youDg to bother about
saving. I should like to say on that point that no one can
be too young to save, and that those who begin to save
?early get the largest return on their money; and they will
find that they can obtain here the comfort of a pension at
?the age they fix to retire, at a rate much less than they
?could ever hope to get it at, or than it would be possible to
get it at elsewhere, and at a rate which they could not
possibly get elsewhere, if they joined immediately they took
their certificates.
The Habit of Saving?Marriage.
It is not a question of the amount of the pension
which you first take out, it is the importance of acquir-
ing the habit of saving. Therefore, if a nurse will only
begin with a small pension?she can take out a policy
for any amount she likes?it would bring her into connec-
tion with the Fund, get her into the habit of saving, and as
her income and resources increased, she would be able to
take oat further policies, and so she might build up that
which must be a great safeguard and comfort to her after-
wards. Here I should like to say this with respect to those
very young nurses who probably think?as I believe many
of them do?that they will one day be married. This Fund
is certainly a premium upon marriage if it is rightly under-
stood, for we find if we turn to our surrenders that 50 per
cent.?that is half?of all the nurses who surrender do so
because they are either going to be married, or because they
are giving up nursing?probably with a view to marriage;
and speaking as a man to women, I am perfectly certain
about this?that if ever there was a time when men desired
to have thrifty and good managers as their helpmates, it is
in the present day. The nurse, Itherefore, who has shown
that she can take care of herself, and that she has a due
regard to what it is necessary for her as a self-respecting
woman to do for herself in her working days, by putting by
in a fund like this, certainly has a premium put upon her in
the matrimonial market, for every man would say, " That is
the kind of woman to look after my house, to make me a
good wife, and if we join hands there is a much better pros-
pect for us both than if I chose a thriftless, extravagent lady
who spent all her earnings on her dress."
Some Objections Considered.
Another objection is that the Post Office Savings Bank
is very much simpler, and that on the whole it is better
for the nurses. Now I want you clearly to understand
this point, and I am going to speak quite as simply
as I possibly can, because I know it is a little diffi-
cult for ladies to understand these matters; but take
the case of a nurse who is 35 years of age, and who
decides to put ?1 in the Savings Bank every month, with
the object of having a sum at her disposal When she is
50 years of age, and she thinks that she may then probably
decide to buy an immediate annuity, in the Post Office or else-
where, which would be the equivalent to the pension we give.
Now a nurse who does that gets on her money per cent, at
present compound interest, so that she has at the end of her
15 years of payments a sum which will amount to ?180 plus
per cent, compound interest. If, however, she put the
money into this Fund, and paid ?1 a month for her policy
premium, the result would be that she would have standing
at her credit in the Fund ?180 when she was 50 years of
age, and in addition?not 2| per cent, interest, which is the
total sum she can get from the Post Office, but she would
have all the interest which has been obtained for her
by the best financial advisers in the City of London upon
the money she has put in the Fund. I have already shown
you we are at present earning on our investments over 4 per
cent. She will in any case have?and this is the important
point?all the bonus additions which come out of the aggre-
gate of profits made in this mutual fund, which is managed
by a council not one of whom is paid, and where the work-
ing expenses are so small that, as I have shown you, under
Bourne's tables?and those are the figures I quoted from
the expenses of management are infinitely smaller than i?
any insurance company. The consequence is that there is
really no comparison so far as advantage goes between the
results which would accrue to a nurse who put her savings
in this Fund and who put them in the Post Office. There is
another point I may mention?that you will not continue
to have 2? per cent, in the Post Office, as the interest there
will be reduced very shortly to 2J per cent. That will make
the advantages of this Fund even greater than they are i?
the conditions which prevail to-day. Another objection is
this, " What is the good," says a nurse, " of ?15 a year? I
would rather starve." A nurse who makes a statement like
March 21, 1903. THE HOSPITAL. Nursing Section. 343
that is not really a thoughtful woman, nor has she ever taken
the trouble to understand the working of this Fund, because
it is so built up that its aim is to secure that every ndrse
shall, at any rate, have an adequate provision in her old age.
And we have, as you know, a Benevolent Fund which is now
confined absolutely to policy-holders, and that Fund at the
present time has upon its books 50 annuitants?50 old
nurses, who, from their age and circumstances, were
unable to put by until this Fund was founded, and who
have found comfort and solace and adequate provision
in their old age because they had confidence in those
"ho founded the Fund and joined for a small policy
"hen the Fund was first started. I would also point
out ? and this especially applies to the older nurses ?
"that if you want a provision the Pension Fund will be a
great help to you, even supposing you take out a small
policy of ?10. We have had an instance lately of a nurse
"ho was permanently incapacitated by an accident, and she
had a policy in this Fund for ?10. The secretary had some
difficulty in persuading her when she joined to take out a
policy for so small a sum, but when her trial came, this
accident befell her, and she was disabled from work, this
policy of ?10 a year enabled steps to be taken to move a
beneficent society (and there are several charities of that
kind in this country), the rules of which provide that can-
didates Bhall not be eligible for an annual grant from its
funds unless they have a secured income of at least ?10 per
annum. The consequence is that through Mr. Dick's services
to this individual nurse and the energy he displayed in
getting this nurse to join the Fund, it has been possible,
"ith the aid of this beneficent society, to give her a pension
"hich is sufficient to leave her in comfort for the rest of
her days.
"Kind Friends."
There is one other objection, which is the most serious
of all?that is, the "kind friends" who come forward and
give all sorts of advice and suggest all sorts of changes in
"the method of investment, and who criticise the returns and
the probable results which will accrue from an investment
in the Fund. I have here quite a dozen instances of
clergymen, solicitors, and other people who have had rela-
tives or friends connected with the Fund, who have gone into
the question and given advice to their sisters or relatives that
?n the whole they would be better off if they took their
snoney out and put it somewhere else. Why I mention this
is because I want every nurse who is approached by a " kind
friend" of this description to get accurately from that friend
"hat she proposes she should do with the money, and to send
on that information to the secretary of the Fund. You will
then have sent to you, by the secretary, a statement which
"ill show you exactly what the Fund is doing with your
tttoney and what your money will ultimately result in for
yourself. You can then compare the two statements, and I
am confident that when you have them before you, not only
"ill you be convinced that it is best for you to remain a
niember of this Fund, but that your friends will be con-
vinced too, and that, like a number of correspondents whose
letters I have here on the table, they will express regret that
from an imperfect knowledge and a hurried desire, possibly,
to show interest in an individual nurse, they were very
nearly doing her a great injury. My last word on that point
is this:?Before you decide to surrender, write frankly to
the secretary what your doubts are. Do have confidence
m the authorities of this Fund. When you look at the
Council, when you realise what I have told you about the
voluntary work which is done for the nurses, it seems to me
(and I think the intelligence of the nursing profession?the
intelligence of the whole world will agree with me) that it is
suicidal for a nurse to run away from this haven of refuge
to run away without thinking, taking her money with her?
frequently to come back later on with sorrow and without
means, to ask if we can give her some help from the Bene-
volent Fund. Before you change your investment take those
precautions which every business man and woman takes,
and ascertain what your present investment is doing for you
in this Fund, and compare the result with anything that is '
offered to you elsewhere?not only as to immediate results,
but permanently, constantly, securely. If you do that you
will do yourself a double service?you will save your money
and get the best return you possibly can on it, while the
Fund provides you as a policy-holder with the power of doing
good among all your sisters wherever you go; and you may
show by the figures and facts that this Fund is what it
really claims to be?the safest, the most remunerative, and
the best society for nurses which there is in the whole world.
The Fund and Hospitals and Institutions which
Employ Nurses. .
I want to say now a few words as to federation. We have a
plan by which we ask institutions to federate with us. They
join the Fund?the committees of the institutions?under
various schemes. It amounts in brief to this: they desire
to do two things?they want to provide that every nurse
who is in their employ shall make such a provision, with
their assistance, that there will be no claim upon the
institution if the nurses become disabled or grow too
old to continue their work. In making that arrangement
they agree to pay half the premium or to make some definite
plan by which they encourage the nurse to save; and so
long as the nurse remains in the institution they go on
paying a proportion of her premium, and in that way they
bind the nurse to the institution and help her to be thrifty
and do good to herself. There is another side to federa-
tion which has an important public aspect, and it is a
matter upon which I wish to dwell for a few minutes. Now-
adays there is a private nursing staff attached to every large
hospital. If a private citizen or a doctor wishes for a
trained nurse he can get that nurse by writing to the matron
of one of the great hospitals, and it is no doubt a most
excellent thing for the public that this arrangement
should prevail, because it is a guarantee that the nurse
who is sent is a woman of character, a woman of knowledge,
that she will be adequate to discharge all the duties de-
volving upon her; and I hope she may prove a friend in
every way in the household she enters. But ought a charit-
able institution to farm out nurses and make a profit out of
the work of the nurses 1 That question has been answered
in the negative by one great institution in London, Guy's
Hospital. They have a scheme of federation with this Fund.'
It is the best of all that has so far been devised to help the '
nurses, and it is one which I am sure will put Guy's Hospital
nurses at a higher premium than those where no such system is
in force. This scheme provides that out of the profit made by
the work of ,Guy's Institution?the private nursing staff?a sum
shall be taken at the discretion of the treasurer and divided'
among the nurses. A nurse who has completed five, or six,
or seven years, or up to nine years' service is entitled for the
period of each years to four shares or five shares or six
shares up to eight shares in the bonus fund. The bonus fund'
consists of the net profits made by the staff of the Guy's Hos-
pital Institution. The institution intimates to us what the
actual value of a share is in a particular year, how many'
shares each nurse is entitled to, and then the institute pur-
chases a policy in the Pension Fund for whatever sum is pro-
duced by each nurse's share, the annuity commencing in the
case of these nurses at 55 years of age. At that age the
policies are all assigned to the nurses. I was talking to Dr.
Perry, the superintendent of Guy's Hospital, about this'
scheme, and he says that after they have worked it, as they"
344 Nursing Section* THE HOSPITAL. M^rch 21, 1903-.
, ROYAL NATIONAL PENSION FUND FOR NURSES?Continued. (fi .
have,now, for spv<pn or eight years, it is perfectly clear that
under it the majority of the nurses on the private staff of
Guy's Hospital will be able to retire with a pension of at least
?50 per annum. That is the sort of scheme which I should
think would commend itself to the responsible managers of
every hospital which has a private nursing staff. What pro-
portion of the profit, whether all or three-fourths, made by
the nursing staff should be divided in this way is a question
for the managers themselves to determine, but I am sure that
the knowledge that such a scheme is in force in a voluntary
institution will not only tend to increase the popularity of
that institution, but it is also calculated materially to
increase its revenue from other sources, because people
who know that the nurses there are adequately provided
for, and that the institution declines to sweat or to make a
profit out of its nursing staff, will be disposed to take a
greater interest in such an institution than they otherwise
would. An institution like this, I believe, is sure to have
tfie whole sympathy of the public behind it, and will obtain
a much larger return in annual subscriptions than those
institutions which neglect this plain duty.
'??????* Thaxks to the Officers.
it is now my pleasant duty to ask you to give your
most hearty thanks to those who have worked for the
Fund. We have first of all to thank Mr. Hambro, the
chairman of the Fund, who is most regular in his attend-
ance, who yields to none of us in his interest in the
work, and who always places his whole time and influence
at the disposal of the nurses through this Fund. I
have further to ask you to thank Mr. Dick, the Secretary of
the Fund, who has been with us now for over ten years, and
under whom the Fund has grown and increased in prosperity.
'Iliose of us who are on the Council know that Mr. Bick's
whole heart is in the work, and that if it were not so
it would have been impossible to have the Fund as effi-
ciently administered as it is, or to have achieved the
satisfactory results we have accomplished. We have also to
thank Lady Rothschild and the ladies associated with her
in the Benevolent Fund, the work of which is most
admirably done. I was talking to the members of the
Council at the last meeting about it, and I said that it was a
pleasure to attend one of their meetings, because of the
thoroughness, the courtesy, and the knowledge brought to
bear on all the cases that arise, and which are increasing
every year. Lady Rothschild has given of herself in full
measure on behalf of the nurses. Since this Fund was
established she has never failed to place her house
at the disposal of the committee for their meetings,
and she has never once been absent from any meeting
on behalf of the Benevolent Fund. I am sure, there-
fore, that you will render your grateful thanks to her
and also to Mrs. Pritchard Binnie, the hon. secretary,
and Mrs. Farmer, the secretary of the Benevolent Fund. If
there are any of the annuitants of the Benevolent Fund
present I am quite sure they would like heartily to thank
Mrs. Farmer, because it is her duty to visit all the annuit-
ants, and, indeed, all applicants under the Benevolent Fund,
and to report on every case before anything is done with it.
She does that work most excellently, and we are very much
indebted to her.
Y . V. ? t' ' ' >?')!' ? . ; ?, ?? .
Message from Her Majesty the Queex.
I have told you that this Fund has grown to such pro-
portions that, it has at present upwards of three-quarters
of a million'in funds, that it has issued over 12,000
policies, and that it' is distributing in pensions every year
?8,0Q0, in addition to the money which is paid away"
through the Benevolent Fund to the 50 annuitants. If, there
is one cause which contributed, perhaps more than any
other in the early days of the Fund to its success, I think
we must attribute it to the courage?the exceptional, the
unusual courage, which was shown by King Edward VII..
and Queen Alexandra. Before this Fund had been in-
corpprated very long the King became the Patron and Queen
Alexandra the President of the Fund; and since that time
they have taken an untiring interest in everything which
has concerned its development. I have received a telegram
from the Queen, to whom I sent, the report and also
the Secretary's report, which her Majesty read. The
telegram is as follows: Queen much pleased with report and
statistics and congratulates you on flourishing condition of
Fund.?Charlotte Knollys." I am quite sure that it will
be a help to the nurses and an encouragement, too,
to them to know that that telegram has come from the
Queen. Whenever the opportunity has arisen Her Majesty
has given her time to receive the nurses and present
them with their certificates, and she is prepared to
continue that practice although she is nthe Queen.
You will realise that in that example every nurse
has reason for encouragement, and it should be a matter as
gratifying to the nurses as it is encouraging and gratifying
to the whole Council of the Fund.
An Example to Hospital Officials.
Her Majesty's example, too, might well be taken to
heart by hospital officials ? chairman, committee men,
matrons, and secretaries, for I take it that it is the
duty of every intelligent man or woman to help those
who are less fortunately circumstanced than themselves;
and a grave responsibility rests upon the managers of every
benevolent institution?and especially of every hospital and
society which employs nurses?to see that they do not fail
to urge on the nurses, and to take personal trouble in bring-
ing before them, the opportunities afforded by a fund like
this, so that every member of their staff and every working
woman in the nursing field whom they touch should at any
rate have every opportunity which they can give, in order
that they maybe enabled to understand what the Fund is
capable of doing, and thus be encouraged, in health, to take
that most difficult step for a woman to take of commencing
to save. It is certainly a very extraordinary thing, but it is a
fact that with the exception of some of the matrons, who are
doing most excellent service which does them honour, most of
the secretaries of these institutions seem to be indifferent as
to whether or not the nurses save. So it has happened
that where hospitals have been federated to the Fund they
do not show very good results in regard to the new policies
which are taken out in the National Pension Fund. I have
made these remarks at the conclusion of my speech to-day
in the hope that they may arouse attention to this matter,
because it is only want of thought, I am sure, which causes
this indifference: and I believe?for I know the whole body
of hospital officials well?that the subject has only to be
mentioned to ensure their thinking about it. It is the high
privilege of the office they hold to take care to make this
Fund known to their staffs, and to encourage every member
of their staffs to look into the matter, and while in health to
commence to save by taking out a policy in this Fund.
(Cheers.)
Mr. Thomas Charles Dewey, F.I.A.: Sir Henry Burdetfc
has asked me to second the'motion for the adoption of the
report, and I have very great pleasure in doing so, although
after the Chairman's exhaustive speech I think there is very^
little for me to add. But I should like to state that I had,
an opportunity a short time since of looking minutely into.
March 21, 1903. THE HOSPITAL. Nursing Section. 345
the rates set out in the prospectus of the Royal National
Pension Fund, and also of referring to the valuation returns
"which have been deposited with the Board of Trade; and I
was convinced that the Pension Fund was in a better posi-
tion to give a better value in the shape of pensions for the
premiums paid than any other commercial undertaking. I
shall be told, probably, by some of those " kind friends " to
whom Sir Henry Burdett has referred, that if you compare
our prospectus with that of the Insurance Department of
the Post Office, or of some other institution, you will find
that the rates, compared with those given in other prospec-
tuses, may be considered high. That I freely admit, but we
dust look a little beyond. The rates that are published by
the Post Office and other institutions will be for the actual
pension that will be paid when the pension matures; there
will be no bonus added; it will be that exact sum. The
figures, however, which are given in the prospectus of the
Pension Fund are really the minimum sums that are guaran-
teed ; in every case there will be an increase to the pension,
and an increased pension arising out of two bonus funds.
There is the bonus that is increasing year by year in con-
sequence of the low rate of expenditure. Last year the
expenditure was 3J per cent., whereas 5 per cent, had been
put by to pay the management expenses. That alone will be
a saving of ?1,500 for the year and the report shows also that
?6,000 per annum is the difference between the interest
earned and the interest assumed in the tables. Those two
items go materially to increase the pensions which will be
paid and in addition we have this exceptional donation
bonus fund at the present time amounting to ?70,000, and
producing in interest ?2,800 a year. By these funds the
pensions must be so considerably augmented as to equal -
111 ore than equal?any pensions that could be given for the
same premiums by any institution on either side of the
Atlantic. Sir Henry Burdett has alluded to what be terms
the savings bank department, which is a very good name
A0r the surrender of policies. I am only sorry the surrenders
are so numerous. At the same time it is an advantage, and
a very great advantage, to persons paying, to know that they
will never rpceive less than they have paid in. But the
other advantage in connection with this Institution?and I
feel that it is the greater advantage?is that we really all are
a federation of fellow-workers in a noble cause and a federa-
tion not in name only. We are all here members of the
same institution, all taking a personal interest in the success
the institution, and in the welfare of every policy-holder.
1 am only astonished that with the exceptional advantages
that are really offered to nurses the number of those who
have taken out policies is so small when compared with the
iarge number of nurses all over the kingdom ; and I should
he delighted if Mr. Dick, who has done so much for this
institution, could find time to go and visit the principal hos-
pitals in the kingdom, and to personally explain to the
nurses the nature of the benefits that are really within their
reach ; for directly the nurses realise what they are losing
?nr policies will be doubled. I have very great pleasure in
seconding the adoption of the report.
The Chairman: Would anyone like to ask any questions ?
Then, before I put the resolution, I should just like to say
that Mr. T. C. Dewey, who has just spoken, is the General
Manager of the Prudential Assurance Company, so be under-
stands all about savings and all about insurance matters,,
and I think that his remarks will have added weight when
you realise that fact. I now put the motion for the adoption
?f the report, and those who are in favour of it will please,
signify the same in the usual way. On the contrary.
C^ied. I have now to caU on Mr. Waley to move the next
resolution. I should like to say that we are much indebted,
to Mr. Waley, because his firm, Messrs. Joseph Sebag and
Company, are kind enough to undertake the valuation
of our securities every year.
Mr. A. J. Waley: Before I move this resolution I should
like to supplement the remarks you have made with refer-
ence to the class of investments held by the Fund. It has
been, as you say, my privilege to make the valuation of the
fands every year, and I confidently assure the policy-holders .
that the investments are well distributed as regards risks,,
well chosen as regards security, and also as regards the rate
of interest they return. Every policy-holder can therefore
feel well assured that the money is in every way securely,
wisely, and properly invested. I now move the re-election -
of the following members of Council, who retire and are
eligible for re-election?Sir John Watney, Mr. Thomas
Bryant, F.R.C.S., Mr. Alfred C. de Rothschild, and Mr.
Herbert P. Hawkins, M.D., F.R.C.P.
Mr. Percival A. Nairne : I second the resolution, Sir.;
? The Chairman put the resolution in the usual way, and
having declared it carried, saidThe Hon. Herbert Gibbs'
will now announce the result of the poll.! '
The Hon. H. Gibbs accordingly read the result as follows:?
We hereby certify that we have examined the ballot cards
received for the voting for the annuitants' and policy-holders'
representatives. 1,401 cards were received, 21 were spoiled
or contained more than seven names. The following is the
result of the votingMiss Mabel Cave, 1,325 votes; Miss
E. Fisher, 1,320; Miss K. H. Monk, 1,337; Miss P. Peter,
1,306; Miss F. Smedley, 1,310; Miss 8. A. Swift, 1,330;
Miss E. Vincent, 1,315; Miss S. Morris, 23; Miss J. E.
Styring, 5; Miss Hamilton, 5; 2 Ladies, 3 each; 3 Ladies,
2 each; 22 Ladies, 1 each. March 12tb, 1903. We are
informed that 5,363 cards were sent out. Herbert C. Gibbs.
Geo. W. Potter.
The Chairman : The cards will be destroyed in accordance
with practice.
Mr. Rawlins: I rise for the purpose of proposing the
re-election of the auditors, Messrs. Whinney, Smith and
Whinney, who have discharged their duties to the full satis-
faction of the Council.
Mr. Charles W. Trotter: I second that.
The Chairman put the motion, and it was carried
unanimously.
Sir Cuthbert Quilter, Bart., M.P.: Sir Henry Burdett,
ladies and gentlemen, when I received an invitation from
our chairman to attend this meeting to-day, I thought it
would be hardly possible for me to be here. I had an engage-
ment in another place, where statements are made equally
promising and equally rosy to those which have been made
here, but the results are not so satisfactory as those to which
we have listened. (Laughter.) I envy Sir Henry Burdett
for having been able to make such a statement from the
chair as that which we have heard; I envy him because I
think that that man is the happiest of all who sees a satis-
factory result of a great work which he has had a large
share in initiating, and it must be most gratifying for him
to think of the immense number of people who have thus been
benefited, especially so as they belong to that sex towards
which we all feel so warmly and owe so much. I am not
accustomed to this sort of meeting; I am a man of business
and have to mix a good deal with the world, but I must say
that I have never in the course of a long career listened to
such a satisfactory statement, regarded from every point o?
view, as that which I have listened to to-day?satisfactory
in regard to the inception of the Fund; satisfactory in the:
class, of men who come forward to lend their great abilities
as well as their great names to an institution of this kind;
satisfactory in the way the business seems to have been
built up, as far as I can judge from the report to-day, step
346 Nursing Section. THE HOSPITAL. Maech 21, 1903.
ROYAL NATIONAL PENSION FUND FOR NURSES?
by step; satisfactory?extraordinarily satisfactory?in the
result to the nurses themselves, and in the wonderful oppor-
tunity that is offered to them for the investment of their savings
and taking out policies to secure them against ill-health
and other circumstances. All I can say is that my mouth
watered when I heard Sir Henry say that, greatly owing to
the people who look after these investments on behalf of
those who can take out policies in this society, a return is
made, I think he said, of over 4 per cent.
The Chairman: ?i 2s. 9d.
Sir Cuthbert Quilter : Where else is it to be done except
here ? These opportunities that are offered to nurses here
are not open to the ordinary individual; they are not open
to the man in the street. All sorts of i inducements, all
sorts of returns for money, more or less problematical, are
open to him, but a safe return of over 4 per cent. I do not
think he could get, and I fay that having had some experi-
ence of this sort of business for a great many years.
Certainly it is a wonderful chance that is offered here to
those who can avail themselves of it, and I am surprised
rather that Sir Henry Burdett and our other good friend
there should have spent so much time in elaborating the
matter. It does not require any great genius to ascertain
that it is far more advantageous to take 4 per cent, in a
Fund like this than 2| per cent, or even 2f per cent, from a
Government Savings Bank. Regret was expressed as to the
withdrawals, but when I heard what the cause was of most
<jf the withdrawals I cannot say that I shared that regret.
It seems to me that the only danger which besets this insti-
tution is lest it should come to the observation of the
Government that it is a great matrimonial institution.
(Laughter.) I hope that that will not be the case, and I
shall take care not to repeat anything I have heard to-day in
that direction, although I must seriously warn Sir Henry
Burdett that he must be a little careful that he does not
place himself within the meshes of the law. (Laughter.) I
was almost forgetting the resolution which I rose to propose,
having been so glad to have this opportunity of congratulat-
ing the chairman on the great results which have been
achieved by this Fund. I trust you will go on as you have
done in the past, atd I would say to those around me, While
these able and distinguished ladies and gentlemen are pre-
pared to continue to work for you gratuitously, as they have
done, take every advantage of it. As your Fund gets built
up year by year, it must get stronger and stronger, and I do
not think you need fear the competition even of his Majesty's
Post Office or of any other institution. I congratulate jou,
Sir Htnry. I remember well your efforts to start this Fund,
and I repeat again that I envy you your success in the
results which have been accomplished?results which I hope
to see continued for many years with your connection with
the Fund. I propose a hearty vote of thanks to Sir Henry
Burdett for presiding.
Mr. F. D. Mocatta : I am very pleased indeed to have the
signal honour conferred on me of being asked to second this
resolution, for I feel overflowing with gratitude for what
has been done here, and I wish to thank Sir Henry Burdett
as far as I can. I have listened with very great attention
to the exhaustive speech of our chairman, and I have learnt
from it a great deal of valuable information. Sir Henry
went over the ground from the beginning to the present
time, and I think that we have all taken in a great number
of fresh ideas from what he has said. It was very gratify-
ing for me to learn that this Fund, which I so much appre-
ciate, ia not only thoroughly solid, but is at the same time
very remunerative; and I think that some of our com-
mercial friends would very probably like to invest in the
Nurses' Fund. ! '
Sir C. Quilter: They would if they had the chance,
(Laughter.)
Mr. Mocatta: It offers very much more than can be got
elsewhere, as well as being very secure indeed. There is.
the great guarantee of some of the best, names in the City
of London, and the rate of interest obtained on the in-
vestments is one which ordinary mortals cannot very easily
get. I think if any class of our society deserves well
of us it is those noble women who devote themselves
to healing sickness and to studying the various details
of hospital treatment. One sees with great pleasure
that the rights of nurses are now very much more
acknowledged than they were a generation ago. Formerly
one used to visit a hospital and to go away rather pained
at the reflection that these noble women who were devoting
themselves so much to works of the highest charity had not
been properly cared for; and the rooms they inhabited and
the course of life they were obliged to live were such as not
to conduce to their own good health or to redeem the debt
of gratitude which society in general owes to nurses. Sir
Henry told us of the efforts which were necessary to induce
nurses to take part in this great privilege of insurance
which is so splendidly carried out by this society. That is
very likely, for you cannot always persuade people to be
thrifty ; and although our nurses generally are very sensible
women, it may occur to them sometimes that it is not a
desirable thing to take measures of thrift when they
are still flourishing and still fairly young. I think
that perhaps the highest form of charity is to
induce thrift, and one of the greatest misfortunes
that society is involved in is that a great number of
people who work very hard neglect putting by some-
thing for a rainy day. I myself, in a very humble capacity
have often had very great difficulty in inducing my friends
who were hard workers and not very wealthy to acknow-
ledge the duty of putting by something for the future. I
am quite sure that where one succeeds in inducing thrift,
one also induces a great deal of happiness in the future and
a great deal of safety for our society. I think sometimes
with sadness?almost with horror?of a great' number of
friends who are making liberal earnings in their various
careers, and who spend every penny they get, and when
some sort of misfortune comes, as it will come upon all
humanity, they are left resourceless and almost in poverty.
I think we may be pretty certain after what we have
heard this afternoon that such a case as that is not
likely to befall our respected friends, the nurses. A
great deal has been done, not only a great deal of hard
work, but a great deal of intelligent work has been
devoted to this subject; and admiring as I do the efforts of
the nurses, to whom we are all so deeply beholden, I also
admire very much the hard work and intelligence which
have been devoted to promoting the welfare of this Society,
which we all know has succeeded so wonderfully well, and
which has 6et such a good example to society in general.
I will not say anything further, because you have heard a
great deal, but I wish I could Bay such good things as yon
have heard from the chairman, and then I would speak for
half an hour, and in that case I do not think you would be
angry with me. (Laughter.) Our most respected chairman
must be very happy this afternoon, because he views the
result of a long career of useful work which has been 8?
wonderfully successful as shown to us now. I only hope
hat Sir Henry and his coadjutors will continue to work in
the way they have been doing for many years to come. I
am very grateful, and I am sure wc are all very grateful to
1<^r Henry Burdett and his coadjutors, and I second this
vote of thanks with the greatest pleasure.
?March 21; 1903. THE HOSPITAL. Nursing Section. 347
Sir C. Quilter: I will now put the resolution which you are
all so longing to agree to. I do not fenow if I am out of
.order, but I would suggest that you deal with this resolu-
tion as we do in the East country, where if we approve a
resolution up goe s the right band and we say aye. If we
do not, up goes the left hand and we say no* (Laughter.)
I propose that the best thanks of the meeting be given to Sir
Henry Burdett for presiding. All those in favour of that
hold up their right hands and say aye. On the contrary.
Carried unanimously.
The Chairman: Sir Cuthbert Quilter, ladies and gentle-
den,?I regard this vote as a vote of thanks to the whole
Council, and in that spirit I am quite sure you have passed
it, and in that spirit we have received it. You cannot dis-
sociate one member of the Council from another. There is
no member of this Council who will yield to any other member
in his interest, his time, and his efforts on behalf of the
nurses. It is purely accidental that I am the chairman^
someone must be chairman; and you will understaud that in
thanking youi very cordially for this hearty vote of thanks?
and telling yon how gratified I am at the splendid success
of this fund?gratified still more as I am by the knowledge
that the robust faith of our Sovereign and Queen Alexandra
has been so abundantly justified?I thank you very heartily
indeed on behalf of my colleagues and myself. I am looking
forward?I hope my life will be spared until that time?
when we shall be able to come here?it cannot be long
distant?and say that the Nurses' Pension Fund investments
reach a million sterling. When that is accomplished I think
I may rest on my oars, and I may then make room for some-
one else to take up this good work. I thank you most
heartily for passing this vote of thanks.
The proceedings then terminated.
?Ibe Burses' JSooftsbelf.
The Midwives' Pocket Book. Second edition. By Miss
Honnor Morten. (The Scientific Press, Limited.
Is. net.)
In Miss Honnor Morten's " Mid wives' Pocket Eook and
uide to the London Obstetrical Society's Examination"
a nurse will find in a small space as much advice, caution,
and valuable guidance as she is likely to need after proper
training in a midwife's duties and obligations. In the
preface to the second edition the author brings the work
*nto line with the new Midwives' Act of 1902, and this
8 ould be carefully read by intending midwives, and not
Passed over as prefaces are apt to be. The obvious advan-
tages of a general training to a midwife are very sensibly
Put forward on p. 2, and a year's training in the general
principles of hygiene, asepsis, and the clinical management
a case would add enormously to the effectiveness of the
id wife and the comfort and well-being of her patients,
timely warning against the prescribing of medicines will
e found on p. 7, and this little work throughout adopts a
se tone of caution to midwives against usurping the duties
qualified practitioners, to their own or the patient's detri-
ent. ip the paragraph on infants' feeding, p. 73, no
ba^? *S m^e tbe value of asses' milk for hand-fed
les. Though from its expense it is beyond the reach of
inf has been undoubtedly the means of saving delicate
p a?ts> and deserves mention. At Welford's Dairies, Edgware
boHl' asse.s are kept and the milk is despatched in sterilised
es to its destination. For infantile hernia, a not uncom-
^ ^^urrehce in newly born children, p. 77, the author
an 1 " ^nd a pad of linen over the place, and get advice
we would add to this, let the midwife's training include
e use and deft application of the wool-skein truss, which,
cu?^e ?PP^ed and kept clean and in position, will help to
re an inguinal hernia in two or three months. We Shall
bej?e see a third edition of this midwives' vade m&fcti'rh
j?ven?6o&\>'s ?ptrtfon.
?Correspondence on all subjects ia invited, but we cannot In anjjr
way be responsible for the opinions expressed by our corre-
spondents. No communication can be entertained if the name
and addresB of the correspondent are not given as a guarantee
of good faith, but not necessarily for publication. All corre-
spondents should write on one side of the paper only.]
""'lTf THE DIET OF CONVALESCENT PATIENTS.
" Hardworking " writes: I sometimes wonder if private
nurses find the same difficulty as I do with the convalescent
patients' diet. So often it is left entirely to the nurse to
order, and after the inevitable sole, chicken, custard pudding
and jelly has been given my troubles seem to begin. Lately,
when I am in big houses and some special dish has been
served up and enjoyed, I send a little message down to the
cook to ask whether she will kindly give me the recipe, so
that I can hand it on to my next case for their benefit. If I
had my days over again, while waiting to be old enough to
train, I should study cookery in all its branches, as it is so
important afterwards, especially to the private nurse; in fact,
I think it is important to every woman, and I should advise
all young girls to take it up, for whatever position in life
they are going to enter afterwards, it will be more than
useful to them.
THE ASYLUM NURSE.
f An Asylum Nurse " from the London County Asylum,
New Southgate, writes: I have read " X. L.'s" remarks in
your columns, and I cannot think that the writer can possibly
have had real experience in asylums?certainly not the
experience to be met with in everyday life here, of which
the picture drawn by the writer in February 7th is a per-
fectly true one, although I am sorry to say so. Moreover,
would anyone with true knowledge of asylums honestly com-
pare them with hospitals where the buildings and surround-
ings are so widely different. Hospital patients, at least, are
sane although helpless. We, too, had our sick and injured,
and it must be borne in mind that invariably the sick and
injured lunatics are most difficult to manage. Yet they
were all saved in the face of such frightful odds. Had such a
calamity broken out in any hospital with a like result, the
staff would have had the heartfelt sympathy of the whole
asylum world. Many officials passed through that terrible
crisis here at the risk of their lives, and it has left silent
marks that will shorten the years of some. One of our
number who helped at that time already lies in her grave.
We can, I think, pass over the slighting remarks made upon
us through that awful period with the feeling that no reflec-
tion was cast upon our honour by the country.
THE APOTHECARIES' HALL CERTIFICATE.
" A Lady Dispenser " writes: I have seen so many
queries re dispensing in The Hospital that I am sure you
will pardon me for giving these facts concerning the
" Apothecaries' Hall" certificate. In some back numbers of
'The Hospital I have noticed that in answer to several
queries as to whether this certificate qualifies one to take a
post as dispenser you say, " No, only as an assistant." But
may I point out that, in the ordinary acceptation of the
word, it does so qualify 1 True, the certificate states that the
holder thereof is qualified to act as an "assistant in com-
pounding and dispensing medicines," but that means as an
assistant to a medical man, which is practically ^ " post as
dispenser." I myself (holding the " Hall" certificate) have
worked for a doctor with a very large practice for the past
fourteen months; and a friend of mine, with the same
qualifications ^s myself, has a post j as jlady-dispenser at one
of the county hospitals; another friend, p.lso with the "Hall"
certificate, being dispenser at a hospital of 80 beds in a
Yorkshire town. I should certainly describe these ladies
.and myself as "holding posts; as dispensers." The next
certificate, i.e. the " Minor,".qualifies one to act as a chemist
and to open a shop for the sale tor drugs. It can only be
taken after the " Hall" hds befenr held f(ft three years.
348 Nursing Section. THE HOSPITAL, March 21, 1903.
appointments.
[No charge is made for announcements under this Head, and we are
always glad to receive, and publish, appointments. Bnt it is
essential that in all cases the school of training should be
given.]'
Drumcondra Hospital Dublin.?Miss Eveleen M. Dane
has been appointed lady superintendent, She was trained
at the Western Infirmary, Glasgow, and the Fountain Hos-
pital, Tooting. She has since been night superintendent at
the General Hospital, Wolverhampton, and the Royal
Halifax Infirmary.
Keighley Union Infirmary.?Miss Bessie Forbes Whyte
has been appointed charge nurse. She was trained at the
Rotherham Hospital, has been charge nurse under the Metro-
politan Asylums Board, and nurse-in-charge of the Isolation
'Hospital, Rotherham. She has also done army nursing at
home and in South Africa.
Liverpool Cancer and Skin Hospital.?Miss Maud
Taylor has been appointed sister. She was trained at the
General Hospital, Bootle, Liverpool, and has since been
sister at the Children's Infirmary, Liverpool. She has also
held other appointments at Rotherham and Halifax.
Orsett Joint Isolation Hospital, Gray's, Essex.?
Miss Fanny Godbehere has been appointed night superin-
tendent. She was trained at Southwark Infirmary, East
Dulwich, and has since been sister in charge of the surgical
and medical wards at Aston Infirmary, Birmingham, and
charge nurse at the Brook Hospital, Shooter's Hill. She
holds the L.O.S. certificate.
Rotherham Hospital and Dispensary.?Miss Wheatley
has been appointed night sister. She was trained at
Lambeth Infirmary, and was afterwards staff nurse. She
has also been a Jubilee district nurse and sister at The
Retreat, York.
Royal Isle op Wight, Ryde.?Miss Amy J. Lander
has been appointed sister of the men's floor. She was
trained at the Queen Victoria Royal Infirmary, Preston,
Lanes., and was afterwards appointed charge nurse of the
operating theatre and out-patient department for six months.
She has also done private nursing in connection with the
same institution.
Sunderland Nursing Institute.?Miss Christina Aldis
has been appointed matron. She was trained at the General
Hospital, Weston-super-Mare, and has since been staff nurse
at the City of London Chest Hospital, charge nurse at the Essex
and Colchester General Hospital, matron at Stratford-on-
Avon Joint Fever Hospital, lady superintendent of the
District Nurses' Home, Ashton-under-Lyne, and lady super-
intendent of the District Nurses' Home, Barry, South Wale?.
She has also done private nursing at Bishop's Stortford,
and temporary work in connection with the Shipley District
Nursing Association.
Union Infirmary, Leeds.?Miss Elizabeth Moss has been
appointed charge nurse. She was trained at the Birmingham
Infirmary.
Wellington House School, Westgatk-on-Sba.?Miss
Ethel M. Heathcote has been appointed matron. She was
trained at the Hospital for Sick Children, Great Ormond
Street, London, and at the Manchester Royal Eye Hospital,
and has also done private nursing.
West Ham and East London Hospital, Stratford.?
Miss Amy Freda Muller, has been appointed sister of the
children's ward, and Mies Violet Pearson staff nurse. They
were both trained at the West Ham and East London
Hospital, and Miss Mailer has since been staff nurse.
Worcester Isolation Hospital?Miss Hughes has
been appointed nurse-matron. She was trained at Worcester
General Infirmary, and has since been charge nurse for
eighteen months at Worcester Isolation Hospital, and night-
superintendent at West Bromwich Infirmary.
2>eatb in ?ur TRanfts.
On Tuesday last, writes a correspondent, the remains of
Miss Florence Jeffery, 24, one of the nursing staff'of the
Mile End Infirmary, were interred at the Tower Hamlets
Cemetery, Bow, amidst manifestations of great regret.
Whilst performing her duties, Miss Jeffery contracted
typhoid fever, and ultimately succumbed to the complaint.
By her kindness and sympathy she had endeared herself,
alike to the patients and the entire staff, and her decease
cast a gloom over the whole of the infirmary. A large
number of wreaths were placed on the coffin of arum lilies,,
violets, etc., sent by the nursing staff, patients, and from
every department of officers in the infirmary. Many o? the
patients' friends were present throughout the service.
41 Gbe Ibospital" Convalescent fun!*.
The Hon. Sec. begs to acknowledge with thanks the
receipt of 2s. Gd. from "A Grateful Tourist" per The
Hospital Travel Editor,
- TRAVEL NOTES AND QUERIES.
Rouen and the Seine (Doubtful).?Certainly you can do it
for ?10 each, with a little care. I should advise your staying four
days only in Rouen. There are a very few pictures in the Musde,
but of no great merit. The churches', Palais de Justice, and Hotel
Bourgth^roude are the principal attractions. Go to Hotel de la
Couronne, close to the Quai du Havre, or to Hotel du Square,
91 Rue Jeanne d'Arc. Pension terms from 6 francs. For the
remaining 13 days, go on to Caudebec by steamer. Hotel de la
Marine, terms 7 francs per day, or, perhaps, less for a staj- of more
than a week. A charming little hotel. There, and up and down;
the Seine, you will see the ways and manners of the peasants*
Read articles in The Hospital for March 25th, 1899, and
August 18th, 1900.
Swiss Tour (M. B.).?The numbers for this tour are complete.
Will other correspondents kindly note this'? There is no charge
unless you want an answer by post; replies in these columns are
quite free.
Nurses' Convalescent Fund (Country Woman). ? Many
thanks for your kind and welcome contribution, and for the many
pleasant things you say of this page. I hope I may have the
pleasure of helping you a third time. The addresses you send are
most useful.
Italian Trip (S. G. R.).?No, references are not required; >t
is enough that you apply through us. Yes, the gentleman
known to me. (2) Sec answer to M. B.
Venice, the Tyrol, and Dolomites (Royal Oak).?It tc^'
be too early for the Stelvio Pass. I have made all inquiries. Tell
me if you want further help. :
Farmhouse Lodgings (E. Wood).?We do not keep lists of
lodgings, but I have two addresses I could give you, one "?
Brighton and another in Somersetshire, both very nice.
these would do, send me a stamped and addressed envelope for
them.
J;9Q3. \THE\-JjOSPITAL. Nursing Section. . 349
Echoes from tbc ?uteifce Morifc.
Movements of Royalty.
Last Friday the first} Court, or evening drawing-room, of
the season was held at Buckingham Palace. The ceremony
began at ten o'clock, and the King and Queen entered the
Throne Room punctually at that hour. Those who passed
before their Majesties remarked that, whereas last year the
King and Queen had stood to receive their visitors, on this
occasion chairs were provided, placed upon a raised plat-
form. This enabled the royal couple to vary the fatiguing
monotony of remaining so long in one position. The Royal
family repaired, after the Court was over, to the Chinese
Room, where supper was served separately for them. For
the guests buffet tables were laid, and nearly a thousand
people were provided for, which included a very large
number of the diplomatic circle. The Queen wore a dress
of white satin, embroidered with gold and precious stones,
with a train]of gold brocade. The Princess of Wales wore
a gown of beautiful ivory white panne. This was embroidered
in a large and effective design with trails of blush roses,
i raised in chiffon from the soft surface of the panne. The
foliage was simulated in various tender shades of green
chenille, and over all small gold paillettes were plentifully
showered. The train, arranged from both shoulders like a
Venetian Court mantle, was of silver lace. Many diamond
, ornaments were worn in the corsage, and the tiara and neck
. ornaments were also of diamonds. The King, it is stated,
proposes to go for a cruise on board the Royal yacht towards
' the end of this month, and will then pay a visit to the King
of Portugal at Lisbon. During his absence the Queen will
1 spend some time at Copenhagen with her father.
The Prince of Wales on Education.
The Prince and Princess of Wales visited Southwark on
Saturday afternoon in order to inaugurate the new buildings
?f St. Saviour's and St. Olave's Grammar School for Girls in
the New Kent Road. The buildings consist of a large
assembly hall, nine class-rooms, chemical and physical
laboratories, lecture, art, music, cookery, and dining-rooms?
there being accommodation for about 300 girls. After prayer
by the Bishop of Southwark, the Princess of Wales declared
the new buildings open, and the Prince in acknowledging a
vote of thanks, congratulated Miss Frodsham, her colleagues,
and the pupils to be instructed by them, upon the excellent
accommodation provided for them. The Prince expressed
bis hope and belief that the training given to the pupils would
be such as to fit them to enter with confidence into the
struggle of life, no matter where their lines might bejcast.
The Tsar's Manifesto.
The Tsar has issued a manifesto to his people which he signed
?n the date of the birthday of the late Tsar, Alexander III.
It has created a very great impression all over his vast king-
dom, and the close resemblance on fundamental points which
it bears to the proclamation which was issued by the late
Emperor in 1881 is a matter of general comment. The
manifesto begins: " On ascending the throne of our ancestors
by the Providence of God, we made a solemn vow before the
Almighty and our conscience sacredly to guard the century-
. old pillars of the Russian power and to dedicate our life to
the service of the beloved fatherland." The Tsar goes on to
announce his decision to strengthen the laws of tolerance
which grant freedom of religion to all his subjects professing
creeds other than the Orthodox faith, and to improve the
conditions of Russian village life and of the local nobility
and peasantry. He indicates the measures to be taken to
obtain this end, among them being a reform of the rural
laws and a releasing and a relieving of the peasants from
the burdens and duties of forced labour.
The Home-coming of Mr. Chamberlain.
On Saturday morning Mr. and Mrs. Chamberlain arrived
at Southampton in the Norman, both looking extremely well.
At Netley a deputation from West Birmingham were allowed
to board the steamer with an address of welcome. At
Southampton, where the Colonial Secretary was welcomed
by the Mayor, a procession was formed to the Hartley Insti-
tute, where an address from the Corporation of Southampton
was presented. Mr. Chamberlain, in the cours e of his reply,
warned his fellow-countrymen not to over-rate the results
which had been actually achieved by his mission; but said
that, although in view of the past, progress towards com-
plete conciliation in South Africa must necessarily be slow,
it would, in his opinion, be certain. He maintained that it
was our duty and policy to concede the political equality
for which we had striven, and, as that would certainly be
done, we might confidently anticipate that, for the first time
in the history of modern South Africa, Dutch and Englisl^
would work together for a common purpose, for the good of
their common country, and for the maintenance of Imperial
interests. Mr. Chamberlain afterwards drove amid cheering
crowds to Southampton West Station, whence he left by
special train for London. At Waterloo he had a splendid
reception from a great gathering, which included the Prime
Minister, and subsequently he was enthusiastically cheered
on his way to his residence in Prince's Gardens. On Sunday
Mr. Chamberlain was received by the King and Queen.
The New Member for Woolwich.
THE election of Mr. Will Crooks as M.P. for Woolwich
affords a remarkable proof of the fact that almost all things
are possible to persons of ability and determination. Mr.
Crooks was in 1861 an inmate of Poplar Workhouse, his
widowed mother, his brother, and three sisters being ordered
in by the Poplar Guardians. Forty years later he became
Chairman of the same Board. By dint of effort and self-
denial, after many rebuffs, he secured a fair wage as a
cooper, and subsequently entered official municipal life as a
trustee and library commissioner for Poplar. In 1892 he
was returned as one of the representatives for Poplar on the
London County Council, and last year he was Mayor of the
Borough, receiving at the close of his year of office an
illuminated address, while Mrs. Crooks was presented with a
gift of plate. He is still Chairman of the Poplar Guardians,
and a Government representative on the Metropolitan
Asylums Board.
Water Colours in Piccadilly.
The Exhibition of the Koyal Institute of Painters in Water
Colours is rich in pictures this year, over GOO works being
arranged at the Gallery in Piccadilly. Naturally, all these
are not worthy of commendation, but a great many are above
the average. Mr. Dudley Hardy's " Toilers," a study of
brawny, weather-beaten fishermen, sorting a plenteous catch
in the sail-furled smack, has considerable merit, but is
unduly large for a water colour. The same fault is apparent
in Mr. Lee Hankey's " It's the Child's Turn Now," a peasant
baby at the base of a tree beside its dead mother. Amongst
the landscapes " Cardross Moss," by James Orrock, " Moon-
rise on the Kennett," and " Clearing after Heavy Weather,"
both by David Green, and " Near Hunstanton," by Claude
Hays, are lall pictures which reflect credit on the artists. Mr.
Aumonier has never done anything better than " Way Across
the Common," and Mr. Arthur C. Bell is at his best in
" Rainy Day in Bruges." Of the flower painters, Mrs. Philip
Hemsley's " Violets" is especially good; and prominent
amongst the 400 naturalists are Miss Ethel Webling and Miss
Lesa Hallam.
350 Nursing Section. THE HOSPITAL. March 21, 1903.
Jfor iRea&ing to tbe Sfcft.
"FEAR NOT, FOR I AM WITH THEE."
Alone ? The God we trnst is on that shore,
The Faithful One whom we have trusted more
In trials and in woes
Than we have trusted those
On whom we leaned most in our earthly strife?
Oh we shall trust Him more in that new life!
r 1 - ? . ? ? ( IT 1 * ; r f ,
Alone ? the God we love is on that shore,
Love not enough, yet whom we love far more,
And whom we've loved all through,
And with a love more true
Than other loves?yet now shall love him more:?
True love of Him begins upon that shore !
So not alone we land upon that shore :
'Twill be as though we had been there before ;
We shall meet more we know
Than we can meet below,
And find our rest like some returning dove,
And be at home at once with our Eternal Love !
Faber.
" Having the desire to depart, and be with Christ; for it is
very far better."?Phil. i. 23, R.V.
To enter the intermediate state is to be with Christ, to be
" present with the Lord," dwelling with Him as in the same
country or city. " To be with Christ is life, and where Christ
is there is His kingdom," says St. Ambrose. It was this that
made St. Paul desire to depart, and this is the hope which he
sets before us too. When the Christian soul departs from this
world he goes to Christ. He goes to his Maker, his Redeemer,
his merciful Saviour; his Lord who has bought him, restored
him, guided him, and now finally saves him. And he goes
consciously. To be with Christ when we depart is to be with
Him without any interposing medium. It is to know that we
know Him, that we love Him, that He accepts us, and that
we shall never lose Him. And he goes at once. " Having a
desire to depart and to be with Christ," says the Apostle; as if
the very moment of departure was also the moment of entering
His presence, as if to depart and to be with Christ were actually
the same thing. So it was with St. Stephen. He saw Christ
when he was dying, and prayed," Lord Jesus, receive my spirit."
So, when he had died, Christ, Whom ? he had seen, received
'his spirit. What a thought is this I The eyes closing now
upon this present world of confusion and strife, and opening
upon the holy calm of the unseen Church; closing upon
suffering and sorrow, and openiDg upon joy that shall not
end; closing on darkness and opening on light; closing-
closing for ever on all the powers of evil and the sense and
presence of sin, and opening upon Him who is Himself
Light and Life, Holiness and Love I? William Maturin.
My soul is strengthened. He Who ever liveth
To those who at His midnight footstool weep,
Shall give unto me, even as " He giveth
To His beloved, sleep;"
And, as the priceless boon in peace I take,
Beneath Thy brooding wings my bed shall be ;
And I will lay me down, sure " when I wake"
Of being " still with Thee."
Sara Palfrey (abridged).
IRotes an& Queries.
The Editor 1b always willing to answer in this column, withoat
any fee, all reasonable questions, as soon as possible.
Bat the following rules must be carefully observed
s. Every communication must be accompanied by the name
and address of the writer.
a. The question must always bear upon nursing, directly at
indirectly.
If an answer is required by letter a fee of half-a-crown must be
enclosed with the note containing the inquiry, and we cannot
undertake to forward letters addressed to correspondents making
inquiries. It is therefore requested that our readers will not
enclose either a stamp or a stamped envelope.
Hospital Training.
(178) Will you kindly tell me if a small hospital of 22 beds,
and maintaining no resident medical officer, can give a certificate
to a probationer after she has finished her two years' course of fever
training??C. C.
The hospital authorities can give a certificate, but it would not
be recognised by the Local Government Board, nor by any high
nursing authority.
Maternity Fees.
(179) Will you kindly tell me if a maternity nurse can claim
full fees for a case, when the confinement has taken place at an
earlier date than expected, and she has uot been sent l'or ??A. B.
It is advisable to provide against such accidents when under-
taking the case; usually half fees are paid.
West Africa.
(180) I should like to go to one of the minor hosoitals on the
West Coast of Africa as trained nurse. Will you kindly tell me to
whom I should apply ??F. C. L.
Apply to the Secretary of the Colonial Nursing Association,
Imperial Institute, S.W.
Home.
(181) Can you kindly give me a list of institutions that would
take two girls, 14 and 17 years of age, in the early stage of phthisis
for the open-air treatment ??District Nurse.
A list of sanatoria appears in" Burdett's Hospitals and Charities.'
The Liverpool Hospital for ConsumDtion has opened a branch
sanatorium for open-air treatment at Delamere Forest, Kingswood,
Cheshire.
Special Training.
(182) I expect to go to India in a few months as a village mid-
wife for a missionary society. As I may be required to assist the
lady doctor, could you give me the name of a hospital where I
could obtain three months' training in preparing patients for opera-
tion, together with some knowledge of administering anaesthetics
and dispensing ? What would the expense be ?
The Hospital for Women, Soho Square, W.C., receives lady pupils
for not less than three months' training at ?13 13s. per quarter.
Laundry provided.
Dispenser.
(183) I am a qualified dispenser, and should be very much obliged
if you could tell me the best way to find a post. 1 should prefer
one in a hospital or convalescent home. I have advertised once,
but received no reply.?C. S.
We know no better means of securing a post than advertising if
your teacher cannot assist you.
Work in Tropics.
(184) I have been advised to write to you. I have lately
arrived from Boston, where I have been trained in the New England
Hospital. The course given there is for two years, special atten-
tion being given to obstetrics. Having been born in Barbados, I
should be glad to obtain work in a tropical climate.?E. C.
The Secretary of the Colonial Nursing Association, Imperial
Institute, would probably b8 able to advise you.
Important Nursing- Textbooks.
"The Nursing Profession : How and where to Train." 2s. net;
2s. 4d. post free.
"A Handbook for Nursea." (New Edition). 5s.net; 5s. 4d.
post free. >
"The Human Body." 5s. post free.
" Ophthalmic Nursing." (New Edition). 3s. 6d. net; 3s. 10d.
post free.
" Gynaecological Nursing." Is. post free.
" Art of Feeding the Invalid." (Popular Edition). Is. 6d. post
free.
" Practical Hints on District Nursing." Is. post free.

				

## Figures and Tables

**Fig. 7. f1:**